# Fatty Degeneration of the Kidneys

**Published:** 1862-07

**Authors:** James P. Ross


### ARTICLE XX.

### FATTY DEGENERATION OF THE KIDNEYS. CARDIAC HYPERTROPHY. DEATH. AUTOPSY.

BY JAMES P. ROSS, M.D.

Read before the Chicago Medical Society.

A. L., aged 27, born in Germany, was admitted into the Chicago City Hospital, Feb. 8, 1860. In Nov. 1859, he firs consulted his family physician, Dr. Ernst Smith. At that time he complained of frequent and severe palpitations of the heart, great mental anxiety, and almost entire loss of sight. The treatment consisted in mild derivations and tr. verat. verid. internally, with the tr. iodine and digitalis externally, over the region of the heart. This treatment was continued for one month, affording considerable relief. At this time, the disease assumed the characteristics of a severe remittent bilious attack. In addition, there were slight convulsions, affecting principally the upper extremities, with mental aberrations; cardiac symptoms somewhat lessened; the urine deposited a reddish sediment, but no trace of albumen. Cold to the head, quinine, and mild laxatives, constituted the principal part of the treatment. During the latter part of January, 1860, another similar attack supervened. At this time, the convulsions assumed an epileptiform character, lasting from 10 to 30 minutes, and recurring as often as once every 4 or 6 hours, with delirium intervening. A second examination of the urine failed to detect any trace of albumen. Feb. 8th, was admitted into the City Hospital.

On admission, the patient was delirious a greater portion of the time; sleepless; countenance suffused; eyes injected; and appetite capricious. The apex of the heart beat between the sixth and seventh ribs, three and a half inches from the sternum; impulse full and diffused; cardiac dulness three inches transversely; heart sounds normal. The urine deposited a reddish sediment, was normal in quantity; sp. gr. 1012°; and free from albumen. Ordered blue mass, aloes, and ipecac every six hours, until free movements of the bowels should be produced. Feb. 12, bowels freely moved; mental derangement somewhat less. Ordered sul. quiniæ, grs. iii.; sul. morphia, gr. ¼, every four hours.

Feb. 14. Constant delirium, with sleeplessness. Ordered sul. ether and laudanum sufficient to quiet the restlessness.

Feb. 16. Delirum less violent; bowels constipated. Ordered a cathartic of castor oil and turpentine, to be followed by iodide of potass, 10 grs. *ter die.*

Feb. 21. Had a slight convulsion, lasting about 10 minutes.

Feb. 23. More rational; sleeps most of the time during the day, and wakeful at night.

Feb. 25. Complains of pain in the head; tightness across the chest; no appetite; bowels costive; no albumen in the urine. Ordered a cathartic.

Feb. 28. Patient rational.

March, 3. Delirium for the last two days. Ordered ext. hyosciam. grs. iii. and prot. iodid. hydrarg. gr. $\frac{1}{4}$, *ter die.*

March 8. Patient rational during the day, delirious at night, and complains of pain in the head. Ordered a blister to the neck, and continue the pills. This treatment was continued until the 21st inst. When he had so far recovered as to be dismissed at his own request.

On the 19th of May—nearly two months after his discharge, he was re-admitted into the Hospital for further treatment. At this time the pulse was 91, and feeble; the countenance waxy in appearance, and puffy; in addition, there was dyspnœa, and expectoration of a reddish brown tenacious sputum; no dulness on percussion; on auscultation, coarse crepitation heard over both lungs, with sibilant and sonorous ronchi. Ordered a cathartic, followed by calomel, ipecac, and bloodroot, every three hours. A blister over the left lung, posteriorly.

May 21. Dyspnœa less; expectoration copious; bowels free.

May 22. Slight delirium; urine pale and scanty. Ordered squills, digitalis, and bloodroot, each one grain, every four hours.

May 23. Delirium less; dyspnœa increased; pulse 108; urine scanty. Continue same treatment, with the addition of one grain of calomel to each powder.

May 24. Convulsions all night. Died comatose at 6 o'clock A.M.

AUTOPSY THIRTY HOURS AFTER DEATH.

*Thorax.*—Old adhesions of the left pleura, the right normal. Left lung crepitant throughout; bronchial mucous membrane of deep red color and thickened. The right lung presented much the same appearance, but in a minor degree. Heart enlarged; weight 16 ounces; walls of left ventricle almost one inch in thickness; valves perfectly normal. *Abdomen.*—Liver large, but healthy. Left kidney atrophied; surface corrugated; cortical substance nearly destroyed. Right kidney normal in size, pale and fatty. Other abdominal organs healthy. *Brain*, normal.

*Remarks.*—In the light which the *post mortem* gives the above case, there is no difficulty in making a correct diagnosis. During the lifetime of the patient, from the facts elicited, no certainty could be arrived at. There was almost an entire absence of symptoms directing the attention to the kidneys as the organs involved. The urine was twice tested with heat and nitric acid before the patient was admitted into the Hospital, and twice afterwards, without showing a trace of albumen.

This case is interesting as affording several points of interest not usually present in this disease. First. The absence of albumen. Second. Absence of dropsy. Third. Cardiac hypertrophy without valvular lesion. Fourth. Persistent mania with occasional blindness.

In the beginning the morbid process in the kidneys was fatty degeneration of the gland cells. This rendered them unfit for the performance of their function—the secretion of the urinary ingredients. And when so much of the secreting structure of the kidneys became involved that blood was not depurated of these ingredients, congestion of the intertubular capillaries ensued. This congestion proceeding backward of the Malpighian bodies gave rise to exudation of serum (containing albumen) into the uriniferous tubes, and at an early period of the affection, albumen probably could have been detected in the urine. But during the progress of the disease, thickening occurred in the walls of the Malpighian bodies, preventing this transudation of serum and consequent detection of albumen. This thickening of the Malpighian bodies not necessarily lessening the flow of water, we had in the above case the normal daily discharge. and as dropsy occurs inversely in proportion to the amount of liquid discharged, we are furnished one reason for its absence. But renal dropsy has other elements entering into its production. A watery condition of the blood,—the retention of morbific materials in the systemic circulation, act as agents in its production. In the above case, no doubt, the presence of the retained urinary excrement tended to the production of dropsy, but so gradually did it occur that a compensating process was established in the centre of the systemic circulation, and its occurrence prevented. It is a well established fact, that obstruction in the lungs produces hypertrophy of the right ventricle; and so may obstruction in the systemic circulation give rise to hypertrophy of the left cavities of the heart.

The mania, blindness, and convulsions will be readily attributed to the poisonous action of the urinary excrement upon the nervous centres. The vertigo and convulsions were occasional, the delirium unusually persistent, continuing for months, and, no doubt, the forcible action of the heart contributed to its production. The nervous system gave the leading symptoms. To review the chain of morbid processes in the case we have:

I. Fatty degeneration of the secreting cells of the kidneys.

II. Retention in the blood of urinary excrement, producing: 1st. Congestion in the kidneys, with the conservative process of thickening of the coats of the vessels, by which the exudation of albumen was impeded; 2d. Obstruction in the systemic capillaries, with the conservative process of cardiac hypertrophy preventing congestion and dropsical effusion.

III. Cardiac hypertrophy and morbid blood combining to produce the delirium, blindness, and convulsions.

